# Resveratrol mitigates hypercholesterolemia exacerbated hyperthermia in chronically heat-stressed rats

**DOI:** 10.14202/vetworld.2019.337-344

**Published:** 2019-02-27

**Authors:** Hosam Al-Tamimi, Amani Al-Dawood, Saddam Awaishesh, Tony Abdalla

**Affiliations:** 1 Department of Animal Science, Faculty of Agriculture, Jordan University of Science and Technology, Irbid, 22110, Jordan; 2 Department of Applied Biology, Faculty of Sciences, Tafila Technical University, Tafila, 66110, Jordan; 3 Department of Nutrition and Food Processing, Faculty of Agriculture, Al-Balqa Applied University, Al-Salt, 19117, Jordan

**Keywords:** heat stress, hypercholesterolemia, rats, resveratrol, thermoregulation

## Abstract

**Background and Aim:**

Hypercholesterolemia (HC) is the major leading cause of cardiovascular disease worldwide. Such atherogenic aberration deeply impacts blood circulation. Resveratrol (R) is a polyphenol that has received attention as a hypolipidemic, antioxidant, and vascular agility advocate. Efficient blood redistribution is a key element in mammalian thermoregulation. We hypothesized that R treatment may aid in mitigating hyperthermic responses under both acute and chronic heat stress (HS) conditions in HC male rats.

**Materials and Methods:**

All rats were initially fitted with miniaturized thermologgers to measure core body temperature (T_core_). With a 2 × 2 factorial arrangement, four groups were randomly allotted, in which half of the animals ingested an HC diet (C+), while the other half ingested a control (C-) diet, throughout the whole study duration of 35 days. Seven rats from each dietary treatment, however, received R (R+; 13 mg/kg BW/day), while the rest received normal saline (R-) for 5 continuous days. All animals were maintained at thermoneutrality (TN; ambient temperature; T_a_=23.15±0.04°C) for a period of 30 continuous days (days 0-29). On day 29, an acute HS (HS; T_a_=35.86±0.37°C; for 9 nocturnal h) was imposed. Then, from day 29, a chronic HS protocol (T_a_=32.28±1.00°C) was maintained until the past day of the trial (day 34), after which blood samples were drawn for analyses of platelet (PL) count, total antioxidant activity (TAO), total cholesterol (TC), triglycerides (TGs), and lipid peroxidation (LP).

**Results:**

Switching animals from TN to HS resulted in abrupt rises in T_core_. The HC diet induced a significant (p<0.01) hyperlipidemia over the control of diet-consuming rats. Interestingly, the hyperthermic response to acute HS was highly pronounced in the rats consuming the C- diet, while the C+ diet exacerbated the chronic HS-induced hyperthermia. Despite failure to improve TAO in the C+ diet, R+ treatment caused a marked (p<0.05) decline in nighttime - hyperthermia in C+ rats, likely by enhancing blood flow to extremities (for heat dissipation) as delineated by drastic downregulations of C+ related rises in PL, TC, TG, and LP (HC diet by R+ interaction; p<0.03).

**Conclusion:**

The hyperthermic response in C- groups was attributed to higher amount of feed intake than those consuming the C+ diet. Yet, the R+ improvement of thermoregulation in the C+ group was likely related to enhancement of vascular hemodynamics. Resveratrol intake mitigated chronic HS-evoked hyperthermia in rats. Such an approach is worthy to follow-up in other mammals and humans.

## Introduction

Cardiovascular aberrations are increasingly becoming world endemic and are yet more pronounced in the developed world. Approximately 29% of annual deaths worldwide are due to cardiovascular disease (CVD). One of many cardiovascular issues is hypercholesterolemia (HC), which occurs when total cholesterol (TC) levels are elevated in the bloodstream. This disorder is widespread and is primarily responsible for major cardiovascular complications, with the subsequent atherosclerosis being the leading cause of death in the US (812,000 deaths in 2008 [1]. By 2020, atherosclerosis is expected to be the main cause of death around the world [2]. Healthy subjects perform physiologically well under environmental stress conditions and are less disposed to complications of their immune system than HC subjects. Typically, HC patients suffer hindrance of optimum laminar blood flow and are hence more prone to ischemia and oxidative stress-induced lipid peroxidation (LP) [3]. One of the most important thermophysiological responses to heat stress (HS) is efficient blood redistribution from splanchnic to subcutaneous vascular beds to increase peripheral perfusion to maximize heat dissipation at the body shell interface.

It is still not clear whether HC muffles peripheral perfusion, especially when maximally needed during thermoregulation under HS [4]. In recent years, resveratrol (a plant extract) has been implicated as an efficient hypocholesterolemic agent, with a high potential in improving microcirculation in mammalian species and boosting the overall antioxidant activity [5].

To date, very limited research tackled the relationship between HC, resveratrol intake, and thermoregulatory efficiency under thermally challenging conditions. Therefore, we aimed to examine the potential improvement of thermoregulatory performance in HC rats by short-term resveratrol treatment.

## Materials and Methods

### Ethical approval

Before starting of this experiment, the protocol was approved by the Animal Care and Use Committee at Jordan University of Science and Technology (ACUC) number 5/2013. The care and use of laboratory animals were strictly in accordance with the guidelines prescribed by ACUC.

### Animals and treatments

Male Wistar rats (n=28) at the age of 56±3 days, weighing 201.9±10.1 g were individually housed in plastic shoebox cages (15 cm × 25 cm × 35 cm) for individual monitoring of feed intake and body weight (BW). Each rat underwent a minor surgical procedure as will be described, to implant an intraperitoneal thermologger that measures core body temperature (T_core_). Rats were allowed a 14-day post-surgical recovery period while maintained at thermoneutrality (TN; T_a_=23.68±0.22°C, RH=29.13±0.21%), before initiation of the trial. Subsequently, the trial ensued (day 0), and the TN exposure (T_a_=23.15±0.04°C, RH=27.04±0.10%) lasted for a period of 30 continuous days. On day 29, an acute HS challenge (T_a_=35.86±0.37°C, RH=19.65±0.83%) for 9 nocturnal h (12:00-09:00 h) was imposed, with T_a_ raised from TN to HS within 60 min. Immediately afterward, a chronic heat wave challenge (T_a_=32.28±1.00°C, RH=23.26±0.23%) was maintained until the past day of the trial (day 34 at 14:00 h). Finally, by the end of the trial, all rats were sacrificed (after deep xylazine and ketamine anesthesia), and blood samples collected through cardiopuncture, on which sera were harvested and stored at −20°C until analyzed. Climatologic parameters (T_a_ and RH) within the animal house were monitored using a biometeorological station (Onset U12-014, HOBO, Onset Computer Corporation, MA, USA) suspended at the room center at an altitude of approximately 1.4 m (rat cages level) for the total duration of the trial (T_a_; RH). Daily light cycle was kept at 12 h dark (19:00-07:00 h) throughout the trial.

Rats were allocated to treatments in a completely randomized design, with a 2 × 2 factorial arrangement of treatments, namely resveratrol (R) and high cholesterol (C). Each treatment group consisted of seven rats (n=7). A control (C-/R-) group consumed the control diet throughout the 35-day trial period (diet formulations are presented in Table-1). A second group (C-/R+) consumed the control diet throughout the trial period and received a dose (by oral gavage) of 13 mg/kg BW/day of purified trans-resveratrol (Santa Cruz Biotechnology, USA) dissolved in sterile isotonic (0.85%) saline solution during the past 5 days of the trial. Each gavage volume was equalized to 3 mL/animal and was gradually provided within 10-15 s. A third group (C+/R-) consumed the C+ diet and given saline solution (R-) during the same period, while the fourth group (C+/R+) consumed the C+ diet and received the R+, similarly. Diet formulations were adopted from previous literature [6]. On mixing (Hoqurt HK-401, Taiwan), each dietary mixture batch of 12 kg was stored in sterile Ziploc bags and refrigerated at 4°C until consumed. Feed intake was monitored daily, while BW was recorded on days 0, 28, and 34 of the trial.

**Table-1:** Diet composition (%, by weight).

Ingredient^a^	Control (C-) diet	Cholesterol (C+) diet
Casein from bovine milk	20.0	20.0
Coconut oil	0.0	25.0
Olive oil	5.0	0.0
Cholesterol	0.0	1.0
Sodium cholate hydrate	0.0	0.5
Sucrose	32.5	48.4
Choline bitartrate	0.2	0.2
DL-Methionine	0.4	0.4
Corn starch	32.4	0.0
α-Cellulose	5.0	0.0
Vitamin mix	1.0	1.0
Mineral mix	3.5	3.5

a Ingredients: Casein from bovine milk (C7078-10KG, SIGMA, USA), coconut oil (Greenfields, Jordan), olive oil (Al-Khairat Farms, Jordan), cholesterol (C75209-500G, SIGMA, USA), sodium cholate hydrate (C1254-100G, SIGMA, USA), sucrose (Al Osra, KSA), choline bitartrate (C1629-500G, SIGMA, USA), DL-methionine (W330108-1KG, SAFC, USA), corn starch (S4126-5KG, SIGMA, USA), α-cellulose (C8002-5KG, SIGMA, USA), vitamin mix (AIN-76A, Dyets Inc., USA), mineral mix (AIN-76, Dyets Inc., USA)

### Surgical procedure

Each rat underwent a minor surgical procedure, in which a miniaturized thermologger (DS1922L, Maxim Integrated, USA) tuned to collect temperature at 11 bit (0.0625°C) resolution was implanted in the intraperitoneal cavity, to record T_core_ as means of evaluating core thermoregulatory performance at 15-min intervals, throughout the trial. Individual rat BW was first recorded for optimization of anesthetic dosages. A xylazine-ketamine mix (xylazine hydrochloride 0.25 mg/Kg BW, Adwia, Jordan; ketamine 0.4 mg/Kg BW, Alfasan, Holland) was injected intraperitoneally. On initiation of deep sedation state (verified by lack of paw pinch reflex), hair of the midline abdominal region was clipped, shaved, and sterilized using alcohol and diluted iodine solution (povidone iodine 1% free iodine, Al-Eiman, Jordan). Each rat was kept at a dorsal recumbency position, to expose the surgical site using a sterile surgical drape, and kept warm on a warming pad during the surgery to avoid deep anesthesia-induced hypothermia. To minimize potential surgery-provoked wound complications, all rats were prophylactically administered a 14-day antibiotic course starting 4 days before surgery (Hipralona Enro-S, Enrofloxacin, Hipra, Spain) in drinking water (10 mg/kg BW). To alleviate post-surgical pain, rats received ibuprofen (10 mg/kg BW; Ibugesic, Dar Al Dawa, Jordan) in drinking water for 5 days, and wound gently rubbed daily by Fucidin (Fusidic Acid, Ph. Eur, 20 mg/g, LEO, Denmark), diluted iodine solution, and hydrogen peroxide solution (10%) whenever needed, during the post-surgical recovery period. All wounds healed up well within 7 days after surgery, without any mentionable complications.

### Hemogramic and metabolic analyses

On the past day of the experiment, all rats were anesthetized (as previously mentioned), and blood collected through cardiopuncture, and sera harvested by centrifugation at 3000 rpm for 10 min and stored at −20°C until analyzed. Blood samples were analyzed (platelet [PL] count, TC, triglycerides [TGs], and total antioxidant activity [TAO]- and LP) (ABX vet pack, 130312w3b, Horiba abx SAS, France; Cholesterol, 313AA; TG 230AA, Biosystem, Barcelona, Spain; Oxiselect total antioxidant capacity assay kit, 0628201304, Cell Biolabs Inc, USA; and TBARS assay kit, 1009055, Cayman Chemical, Michigan, USA), respectively.

### Statistical analysis

Body temperature data were analyzed by split-plot analysis of variance for repeated measures [7] using mixed procedures of statistical analysis system [8]. The main plot consisted of the treatments, while the subplot contained time by treatment interaction. The random variable animal (the experimental unit) within treatment was used as the error term. Two covariance structures were first compared in each analysis, compound symmetry and autoregressive [1]. Then, the covariance structure with the largest Schwarz Bayesian criterion value was used, as formerly described [9]. On the other hand, serum variables and performance data were analyzed by analysis of variance for a completely randomized design and computed using general linear models [8]. Data are presented as least square means±1 standard error. Declaration of significance between means was made whenever the associated p≤0.05.

## Results

### Hemogramic and metabolic parameters

As shown in Table-2, significant (p<0.03) cholesterol by resveratrol interaction was revealed with reference to PL, TC, TG, and TAC, being higher due to the HC diet than the control. Interestingly, treatment with resveratrol (C+/R+) mitigated the elevation in PL as compared to C+/R- counterparts (802.80, 932.20±4.12 × 10^3^/µL, respectively), as well as TG (145.00, 221.17±2.45 mg/dL, respectively). However, resveratrol treatment failed to alleviate the HC effect on TC (420.00, 401.83±1.20 mg/dL for C+/R+ and C+/R-; in that order) and LP (41.30, 42.30±0.47 µM for C+/R+ and C+/R-, respectively). However, no cholesterol by resveratrol interaction (p>0.55) was detected with reference to LP (18.10, 42.30, 13.90, and 41.30±0.47 µM for C-/R-, C+/R-, C-/R+, and C+/R+, respectively). Single-handedly, however, R+ was responsible for a drop in PL, LP, and TC in the control diet-fed groups. Furthermore, resveratrol treatment improved TAC in the control diet-fed rats (281.8, 262.4±1.6 µM CRE for C-/R+ and C-/R-, correspondingly).

**Table-2:** Hematological responses of HC rats treated with resveratrol under HS^e^.

Variable	Treatment^f^					p-value		
	
	C-/R-	C+/R-	C-/R+	C+/R+	SEM^*^	C	R	C*R
PL (×10^3^/μL)	779.00^c^	932.20^a^	760.00^d^	802.80^b^	4.12	0.0001	0.0002	0.0008
TC (mg/dL)	83.80^c^	401.83^b^	72.83^d^	420.00^a^	1.20	0.0001	0.0314	0.0041
TG (mg/dL)	48.20^d^	221.17^a^	69.17^c^	145.00^b^	2.45	0.0001	0.0330	0.0037
TAO (μM CRE)	262.40^c^	296.70^a^	281.80^b^	292.90^a^	1.60	0.0129	0.4283	0.0282
LP (μM)	18.10^b^	42.30^a^	13.90^c^	41.30^a^	0.47	0.0001	0.0163	0.5565

e TN (TN - Ambient temperature; T_a_=23.15±0.04°C) was maintained between days 0 and 28. Then, HS was imposed in two subsequent phases; an acute HS wave (T_a_=35.86±0.37°C for 9 nocturnal h; 00:00-09:00 h) on day 29 of the trial, followed immediately by chronic HS (T_a_=32.28±1.00°C); until the end of the trial (day 34). Blood samples were collected (cardiopuncture) on the last day (day 34) for analyses of PL count, TC, TG (TAO; expressed in terms of CRE), and LP.

f The treatments were HC-inducing diet (C+) and/or R+: C-/R- (control) group fed a control diet throughout the trial. The C+/R- group consumed the HC diet (containing 1% purified cholesterol along with 0.5% cholic acid, with high-fat-based mixture), throughout the trial. The C-/R+was fed the control diet and received a dose of 13 mg/kg BW/day of purified trans-resveratrol by oral gavage during the past 5 days of the trial. The C+/R+group consumed the HC diet and received the R+treatment as explained.

* Most conservative SEM. Row means with different superscripts differ significantly (p<0.05). HS=Heat stress, TC=Total cholesterol, TGs=Triglycerides, TAO=Total antioxidant activity, CRE=Copper-reducing equivalents, HC=Hypercholesterolemia, R+=Resveratrol, SEM=Standard error of mean, TN=Thermoneutrality, PL=Platelet, LP=Lipid peroxidation

### Thermoregulatory responses

**Figure-1:**
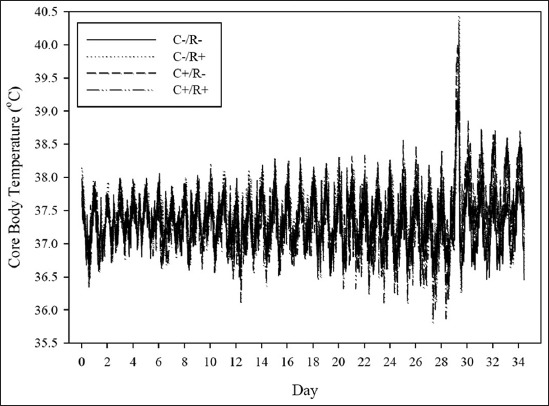
Mean (±SE) core body temperature of rats maintained under thermoneutrality (ambient temperature; T_a_=23.15±0.04°C) was maintained between days 0 and 28. Then, heat stress (HS) was imposed in two subsequent phases; an acute HS wave (T_a_=35.86±0.37°C; 00:00-09:00 h) on day 29 of the trial, followed immediately by chronic HS (T_a_=32.28±1.00°C); until the end of the trial (day 34). The treatments were hypercholesterolemia-inducing (HC) diet (C+) and/or resveratrol (R+): C-/R- (control) group fed a control diet throughout the trial. The C+/R- group consumed the HC diet (containing 1% purified cholesterol along with 0.5% cholic acid, with high-fat-based mixture), throughout the trial. The C-/R+ was fed the control diet and received a dose of 13 mg/kg BW/day of purified trans-resveratrol by oral gavage during the past 5 days of the trial. The C+/R+ group consumed the HC diet and received the R+ treatment as explained.

**Figure-2:**
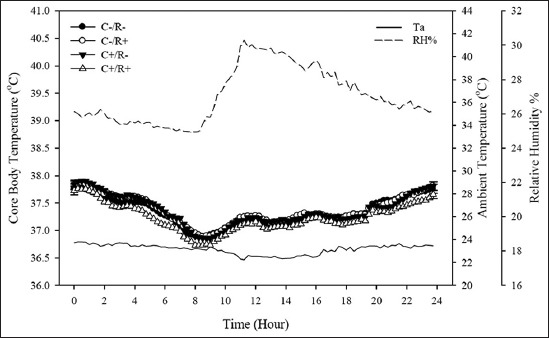
Daily mean (±SE) core body temperature of rats while under thermoneutral conditions. Thermoneutrality (ambient temperature; T_a_=23.15±0.04°C) was maintained between days 0 and 28. Then, heat stress (HS) was imposed in two subsequent phases; an acute HS wave (T_a_=35.86±0.37°C; 00:00-09:00 h) on day 29 of the trial, followed immediately by chronic HS (T_a_=32.28±1.00°C); until the end of the trial (day 34). The treatments were hypercholesterolemia-inducing (HC) diet (C+) and/or resveratrol (R+): C-/R- (control) group fed a control diet throughout the trial. The C+/R- group consumed the HC diet (containing 1% purified cholesterol along with 0.5% cholic acid, with high-fat-based mixture), throughout the trial. The C-/R+ was fed the control diet and received a dose of 13 mg/kg BW/day of purified trans-resveratrol by oral gavage during the past 5 days of the trial. The C+/R+ group consumed the HC diet and received the R+ treatment as explained. No treatment by time interaction was found (p>0.05).

A total of 3360 T_core_ readings per freely moving animal were recorded, without any mentionable disturbances in well-being throughout the trial (Figure-1). No intertreatment variations in T_core_ were detected when the rats were kept under TN (Figure-2), with a pooled value of 37.33±0.06°C. A clear circadian rhythm (time effect; p<0.05) was evident in all rats such that T_core_ was biphasically cyclical, with a noticeable rise coinciding with the daily handling times (10:00-12:00 h and 20:00-24:00 h), similarly in all groups alike.

**Figure-3:**
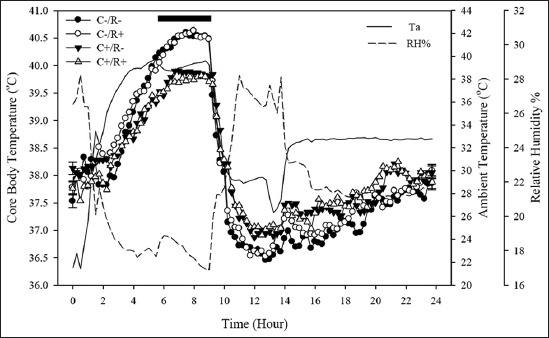
Mean (±SE) core body temperature of rats while under acute heat stress (HS) challenge (T_a_=35.86±0.37°C; 00:00-09:00 h) on day 29 of trial. Thermoneutrality (ambient temperature; T_a_=23.15±0.04°C) was maintained between days 0 and 28. Then, HS was imposed in two subsequent phases; an acute HS wave on day 29 of the trial, followed immediately by chronic HS (T_a_=32.28±1.00°C); until the end of the trial (day 34). The treatments were hypercholesterolemia-inducing (HC) diet (C+) and/or resveratrol (R+): C-/R- (control) group fed a control diet throughout the trial. The C+/R- group consumed the HC diet (containing 1% purified cholesterol along with 0.5% cholic acid, with high-fat-based mixture), throughout the trial. The C-/R+ was fed the control diet and received a dose of 13 mg/kg BW/day of purified trans-resveratrol by oral gavage during the past 5 days of the trial. The C+/R+ group consumed the HC diet and received the R+ treatment as explained. Horizontal bar signifies a cholesterol by time interaction (p<0.05).

On day 29, the acute HS regimen was implemented (Figure-3). The time lapse for the rise from TN to a stabilized T_a_ at HS took 4 h. Animals from all groups displayed a pronounced hyperthermic response. Unexpectedly, however, both rat groups consuming the C- diet (C-/R- and C-/R+) displayed more exaggerated T_core_ rises (0.57±0.06°C; particularly, within the last 160 min of the acute HS) than the C+ groups (C+/R- and C+/R+), with pooled values of 39.42 and 38.85±0.06°C, respectively (cholesterol by time interaction; p<0.01). Subsequently, with the subsiding T_a_ (a 9.98°C transitional drop), T_core_ of all groups precipitously fell down by 2.76±0.06°C within 180 min.

**Figure-4:**
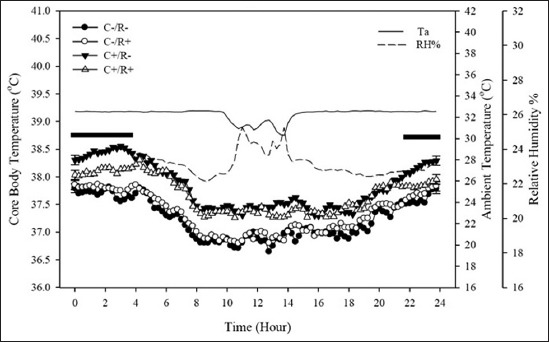
Daily mean (±SE) core body temperature of rats while maintained under chronic heat stress (HS) challenge (T_a_=32.28±1.00°C) between days 29 and 34. Thermoneutrality (ambient temperature; T_a_=23.15±0.04°C) was maintained between days 0 and 28. Then, HS was imposed in two subsequent phases; an acute HS wave (T_a_=35.86±0.37°C; 00:00-09:00 h) on day 29 of the trial, followed immediately by chronic HS. The treatments were hypercholesterolemia-inducing (HC) diet (C+) and/or resveratrol (R+): C-/R- (control) group fed a control diet throughout the trial. The C+/R- group consumed the HC diet (containing 1% purified cholesterol along with 0.5% cholic acid, with high-fat-based mixture), throughout the trial. The C-/R+ was fed the control diet and received a dose of 13 mg/kg BW/day of purified trans-resveratrol by oral gavage during the past 5 days of the trial. The C+/R+ group consumed the HC diet and received the R+ treatment as explained. Horizontal bars signify cholesterol by resveratrol by time interactions (p<0.05).

Succession from the acute to chronic HS (day 29 at 09:00 h on forward) revealed another notable intertreatment T_core_ response (Figure-4). Inversely from the previous acute HS response, the C+ diet-fed rats exhibited a further (0.45±0.04°C; p=0.03) augmented hyperthermic reaction than the C- diet-consuming counterparts (pooled T_core_ of 37.77 and 37.32±0.04°C, respectively). Remarkably, nonetheless, resveratrol treatment prompted a marked alleviation of chronic HS-provoked hyperthermia in the rats consuming the C+ diet (C by R by time interaction; p<0.05), explicitly during the late nocturnal hours (22:00-03:45 h). This resveratrol-evoked mitigation (p<0.05) of hyperthermy in the C+/R+ resulted in 0.35±0.04°C below that of the C+/R- group within the stated late night hours.

### BW and feed intake responses

On the very 1^st^ day of the study (day 0), animals from all four groups had similar (p>0.32) BW (225.71±8.56 g, pooled; Table-3). However, by day 29, the control diet-consuming rats (C-/R- and C-/R+) ingested significantly (p=0.007) more (64%) average daily feed (ADFI) than the two C+ groups throughout the TN period. By day 29, BW of animals on the C- diet ended up having greater BW by 17%. Although ADFI levels did not vary (p>0.15) among treatment groups during the HS periods, the C+/R- rats were the only one to gain weight within the past 5 days of the trial (p<0.04; simple effects of C and R).

**Table-3:** BW, feed intake, and weight gain of HC rats treated with resveratrol under HS^e^.

Variable	Treatment^f^					p-value		
	
	C-/R-	C+/R-	C-/R+	C+/R+	SEM^*^	C	R	C*R
BW (g)								
Day 0	227.14	222.14	232.85	220.71	8.56	0.328	0.805	0.681
Day 29	330.00^a^	279.29^b^	330.86^a^	283.28^b^	10.26	<0.001	0.821	0.884
Day 34	322.86^a^	285.00^b^	322.29^a^	276.29^b^	9.93	<0.001	0.644	0.685
ADFI (g):								
TN	20.89^a^	12.46^b^	18.69^a^	11.63^b^	0.89	0.007	0.670	0.732
HS	17.09	12.69	15.59	15.98	0.76	0.168	0.151	0.540
BW gain %:								
TN	45.61^a^	25.94^b^	42.17^a^	28.41^b^	2.05	<0.001	0.950	0.266
HS	−2.07^b^	2.16^a^	−2.54^b^	−2.41^b^	0.98	0.031	0.034	0.085

e TN (TN - ambient temperature; T_a_=23.15±0.04°C) was maintained between days 0 and 28. Then, HS was imposed in two subsequent phases; an acute HS wave (T_a_=35.86±0.37°C for 9 nocturnal h; 00:00-09:00 h) on day 29 of the trial, followed immediately by chronic HS (T_a_=32.28±1.00°C); until the end of the trial (day 34). Variables measured were: BW, on days 0, 29, and 34; (ADFI; extrapolated from daily measurements; offered - refusals).

f The treatments were HC-inducing diet (C+) and/or R+: C-/R- (control) group fed a control diet throughout the trial. The C+/R- group consumed the HC diet (containing 1% purified cholesterol along with 0.5% cholic acid, with high-fat-based mixture), throughout the trial. The C-/R+was fed the control diet and received a dose of 13 mg/kg BW/day of purified trans-resveratrol by oral gavage during the past 5 days of the trial. The C+/R+group consumed the HC diet and received the R+treatment as explained.

* Most conservative SEM. Row means with different superscripts differ significantly (p<0.05). TN=Thermoneutrality, HS=Heat stress, BW=Body weight, ADFI=Average daily feed intake, HC=Hypercholesterolemia, R+=Resveratrol, SEM=Standard error of mean.

## Discussion

### Hemogramic and metabolic parameters

Higher cholesterol levels have been linked to many health-compromising risk factors such as the development of peripheral vascular disease, CVD, and coronary artery disease [10]. Several studies demonstrated that resveratrol - a natural polyphenol - has protective effects on the circulatory vasculature against vascular lesions [11,12]. Such advantageous effects are primarily mediated by acting as a potent antioxidant and, thus, combating reactive oxygen species and other atherosclerotic substances such as cholesterol and, therefore, reducing PL aggregation and oxidation of low-density lipoprotein (LDL). Hyperlipidemia is well known to impose deleterious effects on the interior vascular lining (tunica intima), primarily characterized by plaque formation and microvascular lesions, which deeply suppresses the immune system to attack or engulf the adhesive atheroma (oxidized LDL) from the vascular walls [13]. Consistent with other studies [14] done on rats fed comparable HC provoking diet for a period of 4 weeks, the HC-inducing diet used in our present trial also triggered remarkable (p<0.01) HC. The chronic combination of dietary cholate (cholic acid) with high cholesterol has been directly linked to HC, chiefly through the activation of adhesive and pro-inflammatory molecules and enzymes (namely, thromboxane A_2_ and cyclooxygenases 1 and 2 [15]).

The current results showed, however, that R+ treatment significantly reduced (p<0.05) TG levels when combined to the HC diet (C+/R+ rats) but failed to reduce TC levels. Contradicting findings with regard to TC responses have also been reported by others [14,15]. These discrepancies may have been a result of time length for resveratrol administration, likely being capable of TC reduction only when applied for longer periods (surely >5 days) [16]. More interestingly, our current data show that R+ treatment successfully alleviated (p<0.01) the HC-induced rises in LP, as well as in the C-/R+ group. Increases in LP by HC have been established before [17]. It is well known that high dietary saturated fat contents induce oxidative stress and flare peroxidation reaction [18]. It is believed that resveratrol enhancement of antiperoxidative stress is attained through reversal of amyloid β protein-induced malondialdehyde overproduction - a prominent biomarker of oxidative stress -induced LP [19].

Furthermore, HS alone has been found to induce both the potential of oxidative stress and/or LP [20]. In shadows of the fact that HC solitarily worsens LP. In the current present trial, resveratrol treatment was not sufficient in lowering LP. It is plausible to suggest that lipid peroxidation with the combination of HC and HS together may be overwhelmingly high to the point that any beneficial effect (antiperoxidative activity) of resveratrol may have been muffled. Furthermore, the relatively short period of R+ application has not probably been satisfactory for that purpose. This proposition may be backed up by recent work in humans [21], suggesting similar reasoning, in which excessive hyperthermia-induced LP can mask the antiperoxidation benefits of antioxidants when HC is chronic.

In general, short-term states of prevailing oxidative stress elicit defensive increases in TAO. Despite the significant rise in TAO in the C-/R+ animals over the C-/R- group, R+ failed to instigate such an improvement in the C+/R+ rats (C by R interaction; p<0.03). In addition to HS-evoked stimulation of protective TAO, HC also activates equivalent rises. The absence of an added favorable R+ effect on the C+/R+ group could be related to a possible maximization of TAO triggered by both collaborative stressors (HS and HC) already.

Usually, TAO is elevated when oxidative stress states prevail and when LP by-products are high, it would be reasonable to postulate that HC diet resulted in a significant (p<0.05) rise in TAC with regard to this research, and resveratrol treatment significantly (p<0.05) increased TAC in the C-/R+ group as compared to controls in the HS trial. In contrast to our findings, rats fed HC diet showed a reduction in total antioxidant status [17]. Contrary, another report is in concordance with our current work, whereby R+ was successful in redeeming TAO in hyperlipidemic rats [14]. It is worthy to point out that the TAO assay used here is non-specific and does not cover the overall antioxidant potential of many antioxidant systems in the body [22]. More comprehensive screening of other specific antioxidants may be needed to elucidate this inconsistency.

### Thermoregulation

As in all homeothermic mammals, observable daily fluctuations in T_core_ were evident in all rats throughout the trial. This repetitive cyclic oscillation (circadian rhythm) corresponds to both, surrounding thermal environment and more importantly to the animal’s biological clock which crosses talks with the ambient light cycle [23]. Moreover, handling of animals, normal laboratory procedures, and even the presence of personnel near the animal alter T_core_ patterns [24]. The current study shows that T_core_ of all animals displayed biphasic forms, whereby a gradual elevation in T_core_ of all rats occurred past the 19:00 h mark, with a rebound back again after 02:00 h (Figures-2 and 4).

When subjected to acute HS, T_core_ of all animals arose significantly (p<0.01), with reference to their TN baseline values. The range of T_a_ where metabolic rate is minimal and body temperature is regulated primarily through the modulation of peripheral vasomotor tone and the concomitant control of dry or sensible heat loss is termed the thermoneutral zone. As T_a_ rises above the TN, heat influx outweighs heat loss, and therefore, T_core_ increases concomitantly under HS. Gradual rises in evaporative heat loss also occur in response to HS, both physiologically and behaviorally. The T_a_ above which evaporative heat loss increases significantly above its equivalent TN level is referred to as the upper critical temperature and was estimated to be 31-32°C in rats [25]. Normal body temperature (normothermia) in rats is generally maintained around 37.5°C [26], while a T_core_ approximating 40.5°C is considered a critical threshold for morbidity and mortality [27]. In the present study, as shown in Figure-4, the T_core_ of C-/R+ and C-/R- was profoundly higher (p<0.01) than in the HC groups when momentarily exposed to acute HS. This magnified hyperthermic reaction can be superimposed onto - and related to - the greater ADFI throughout the TN up until the end of day 28, which links to thermogenic effect of feed just preceding the momentary HS bout on day 29. In fact, the C-/R- and C-/R+ groups consumed 64% more daily feed as compared to their HC counterparts during the TN period. Higher dietary/caloric intake correlates directly to greater postprandial thermogenicity and manifests in higher T_core_ of rats [28]. It is worthwhile to emphasize that T_core_ in all animals dropped sharply within 3 h when HS was switched from the acute to the chronic protocol. Interestingly, this sharp (and yet, transitory) decline drew T_core_ values to equivalent levels previously observed while under the TN climate, likely due to ongoing thermolytic inertia (continuation of a heightened propensity to sharply dissipate body heat load due to being subjected to high T_a_ while under acute HS). Similar findings have been previously reported by our group [29].

On the contrary to acute HS responses in T_core_, the C+ diet aggravated the hyperthermic response in both rat groups (C+/R- and C+/R+; Figure-4), particularly during the daytime. With the absence of sweating-dependent cutaneous evaporative heat loss, rats rely copiously on radiative, convective, and conductive heat loss through the employment of peripheral vasodilation of the ventral vein of tail, to dissipate augmenting heat load in the deep core body sites [24,25]. Consequently, any compromise in the ability to reroute splanchnic blood flow to body extremities under HS will definitely exacerbate hyperthermia in rats [29]. Compelling evidence shows that peripheral perfusion is greatly sub-optimized in HC conditions [30,31], mostly due to hindered laminar flow as a consequence of vascular endothelial injury. Likewise, thermoregulatory failures had been addressed in cases of atherosclerosis and hyperlipidemia, partly for the same aforementioned reasons [32,33]. Recently [34], resveratrol was found to endow optimal perfusion in microcirculation sites and, therefore, was linked to normalizations of systemic blood pressure as a whole in HC patients. Accordingly, we suggested that possible hindrance in blood flow and agility in blood redistribution from the deep splanchnic to peripheral vascular beds may be a predisposing factor in blunting efficient heat dissipating extent at the body surfaces in the present trial and, thus, manifested as intensified hyperthermia. In addition, resveratrol treatment (R+) showed a partially relieving effect reflected as a significantly lower (p<0.05) T_core_ in the C+/R+ animals compared to the C+/R- group. It is thus commendable to assume that due to its hypolipidemic effect, resveratrol may have played a role in partially (nighttime) relieving the hyperthermic intensity observed in the C+/R+ animals exposed to chronic HS. Knowing that HS suppresses appetite [24,29], and in light of the lack of ADFI variations among the experimental groups during the late HS phase, a role of prandial thermogenicity can be ruled out. The improvement in circulatory flow dynamics at the body perimeter by R+ and concomitant enhancement for heat loss stands out to conceivably explain the context of nighttime hyperthermia alleviation.

## Conclusion

The HC hinders thermophysiological responses to chronic (but not acute) HS challenge in rats, likely by impacting cardiovascular hemodynamics. Resveratrol administration aided in the alleviation of nighttime hyperthermia in HC rats under chronic HS and improved several lipid profile and immunological parameters. Such responses are worthy of investigation in humans and other mammals.

## Authors’ Contributions

HA: The full experimental idea, planning, and design. Pre- and post-surgical procedures as well as the surgeries. Setting up all equipment and also carried out all statistical analyses and explained interpretations. AA: Participated in the manuscript writing. SA: Manuscript writing and revision. TA: Preparation of equipment, data collection, and spreadsheet preparations and also participated in the manuscript writing and revision. All authors read and approved the final manuscript.

## References

[1] Kenny G.P, Yardley J, Brown C, Sigal R.J, Jay O (2010). Heat stress in older individuals and patients with common chronic diseases. CMAJ.

[2] Atkins J.L, Whincup P.H, Morris R.W, Lennon L.T, Papacosta O, Wannamethee S.G (2014). High diet quality is associated with a lower risk of cardiovascular disease and all-cause mortality in older men. J. Nutr.

[3] Chatterjee S, Fisher A.B (2014). Mechanotransduction in the endothelium:Role of membrane proteins and reactive oxygen species in sensing, transduction, and transmission of the signal with altered blood flow. Antioxid. Redox Signal.

[4] Rim S.J, Leong-Poi H, Lindner J.R, Wei K, Fisher N.G, Kaul S (2001). Decrease in coronary blood flow reserve during hyperlipidemia is secondary to an increase in blood viscosity. Circulation.

[5] Ficarra S, Tellone E, Pirolli D, Russo A, Barreca D, Galtieri A, Giardina B, Gavezzotti P, Riva S, De Rosa M.C (2016). Insights into the properties of the two enantiomers of trans-δ-viniferin, a resveratrol derivative:Antioxidant activity, biochemical and molecular modeling studies of its interactions with hemoglobin. Mol. Biosyst.

[6] Zulet M.A, Barber A, Garcin H, Higueret P, Martinez J.A (1999). Alterations in carbohydrate and lipid metabolism induced by a diet rich in coconut oil and cholesterol in a rat model. J. Am. Coll. Nutr.

[7] Gill J, Hafs H (1971). Analysis of repeated measurements of animals. J. Anim. Sci.

[8] SAS (2014). SAS User's Guide:Statistics.

[9] Littell R.C, Henry P.R, Ammerman C.B (1998). Statistical analysis of repeated measures data using SAS procedures. J. Anim. Sci.

[10] Mundal L, Igland J, Ose L, Holven K.B, Veierød M.B, Leren T.P, Retterstøl K (2017). Cardiovascular disease mortality in patients with genetically verified familial hypercholesterolemia in Norway during 1992-2013. Eur. J. Prev. Cardiol.

[11] Fauconneau B, Waffo-Teguo P, Huguet F, Barrier L, Decendit A, Merillon J.M (1997). Comparative study of radical scavenger and antioxidant properties of phenolic compounds from *Vitis vinifera* cell cultures using *in vitro* tests. Life Sci.

[12] Frankel E.N, Waterhouse A.L, Kinsella J.E (1993). Inhibition of human LDL oxidation by resveratrol. Lancet.

[13] Sato K, Shirai R, Yamaguchi M, Yamashita T, Shibata K, Okano T, Mori Y, Matsuyama T.A, Ishibashi-Ueda H, Hirano T (2018). Anti-Atherogenic effects of vaspin on human aortic smooth muscle cell/macrophage responses and hyperlipidemic mouse plaque phenotype. Int. J. Mol. Sci.

[14] Zhu L, Luo X, Jin Z (2008). Effect of resveratrol on serum and liver lipid profile and antioxidant activity in hyperlipidemia rats. Asian-Australas. J. Anim. Sci.

[15] González-Peña D, Checa A, de Ancos B, Wheelock C.E, Sánchez-Moreno C (2017). New insights into the effects of onion consumption on lipid mediators using a diet-induced model of hypercholesterolemia. Redox Biol.

[16] Jimoh A, Tanko Y, Ayo J, Ahmed A, Mohammed A (2018). Resveratrol increases serum adiponectin level and decreases leptin and insulin level in an experimental model of hypercholesterolemia. Pathophysiology.

[17] Beltowski J, Wojcicka G, Gorny D, Marciniak A (2000). The effect of dietary-induced obesity on lipid peroxidation, antioxidant enzymes and total plasma antioxidant capacity. J. Physiol. Pharmacol.

[18] Hulbert A.J, Martin N, Else P.L (2017). Lipid peroxidation and animal longevity. Lipid Peroxidation:Inhibition, Effects and Mechanisms.

[19] Huang T.C, Lu K.T, Wo Y.Y, Wu Y.J, Yang Y.L (2011). Resveratrol protects rats from abeta-induced neurotoxicity by the reduction of iNOS expression and lipid peroxidation. PloS One.

[20] Belhadj S.I, Najar T, Ghram A, Dabbebi H, Ben M.M, Abdrabbah M (2014). Reactive oxygen species, heat stress and oxidative-induced mitochondrial damage. A review. Int. J. Hyperthermia.

[21] Streja E, Streja D.A, Soohoo M, Kleine C.E, Hsiung J.T, Park C, Moradi H (2018). Precision medicine and personalized management of lipoprotein and lipid disorders in chronic and end-stage kidney disease. Semin. Nephrol.

[22] Mohammadi M, Alipour M, Alipour M, Vatankhah A (2006). Effects of high cholesterol diet and parallel chronic exercise on erythrocyte primary antioxidant enzymes and plasma total antioxidant capacity in dutch rabbits. Int. J. Endocrinol. Metab.

[23] Goh G.H, Mark P.J, Maloney S.K (2016). Altered energy intake and the amplitude of the body temperature rhythm are associated with changes in phase, but not amplitude, of clock gene expression in the rat suprachiasmatic nucleus *in vivo*. Chronobiol. Int.

[24] Gordon C.J (1990). Thermal biology of the laboratory rat. Physiol. Behav.

[25] Gordon C.J (1993). Twenty-four hour rhythms of selected ambient temperature in rat and hamster. Physiol. Behav.

[26] Dangarembizi R, Erlwanger K.H, Mitchell D, Hetem R.S, Madziva M.T, Harden L.M (2017). Measurement of body temperature in normothermic and febrile rats:Limitations of using rectal thermometry. Physiol. Behav.

[27] Guzmán-Ruiz M.A, Ramirez-Corona A, Guerrero-Vargas N.N, Sabath E, Ramirez-Plascencia O.D, Fuentes-Romero R, León-Mercado L.A, Sigales M.B, Escobar C, Buijs R.M (2015). Role of the suprachiasmatic and arcuate nuclei in diurnal temperature regulation in the rat. J. Neurosci.

[28] Henry B.A, Blache D, Rao A, Clarke I.J, Maloney S.K (2010). Disparate effects of feeding on core body and adipose tissue temperatures in animals selectively bred for nervous or calm temperament. Am. J. Physiol. Regul. Integr. Comp. Physiol.

[29] Al-Tamimi H, Eichen P, Rottinghaus G, Spiers D (2007). Nitric oxide supplementation alleviates hyperthermia induced by intake of ergopeptine alkaloids during chronic heat stress. J. Therm. Biol.

[30] Clough G.F, Kuliga K.Z, Chipperfield A.J (2017). Flow motion dynamics of microvascular blood flow and oxygenation:Evidence of adaptive changes in obesity and Type 2 diabetes mellitus/insulin resistance. Microcirculation.

[31] Zhao G, Etherton T.D, Martin K.R, Gillies P.J, West S.G, Kris-Etherton P.M (2007). Dietary α-linolenic acid inhibits proinflammatory cytokine production by peripheral blood mononuclear cells in hypercholesterolemic subjects. Am. J. Clin. Nutr.

[32] Leon L.R, Bouchama A (2015). Heatstroke. Compr. Physiol.

[33] Csont T, Balogh G, Csonka C, Boros I, Horvath I, Vigh L, Ferdinandy P (2002). Hyperlipidemia induced by high cholesterol diet inhibits heat shock response in rat hearts. Biochem. Biophys. Res. Commun.

[34] Joris P, Mensink R, Adam T, Liu T (2018). Cerebral blood flow measurements in adults:A review on the effects of dietary factors and exercise. Nutrients.

